# 17β‐estradiol reduces SARS‐CoV‐2 infection in vitro

**DOI:** 10.14814/phy2.14707

**Published:** 2021-01-19

**Authors:** Robertha Mariana Rodrigues Lemes, Angelica Jardim Costa, Cynthia Silva Bartolomeo, Taysa Bervian Bassani, Michelle Sayuri Nishino, Gustavo Jose da Silva Pereira, Soraya Soubhi Smaili, Rui Monteiro de Barros Maciel, Carla Torres Braconi, Edgar Ferreira da Cruz, Ana Lopez Ramirez, Juliana Terzi Maricatto, Luiz Mario Ramos Janini, Carla Máximo Prado, Roberta Sessa Stilhano, Rodrigo Portes Ureshino

**Affiliations:** ^1^ Department of Biological Sciences Universidade Federal de São Paulo Diadema SP Brazil; ^2^ Laboratory of Molecular and Translational Endocrinology Escola Paulista de Medicina Universidade Federal de São Paulo São Paulo SP Brazil; ^3^ Department of Pharmacology Escola Paulista de Medicina Universidade Federal de São Paulo São Paulo SP Brazil; ^4^ Department of Physiological Sciences Faculdade de Ciências Médicas da Santa Casa de São Paulo São Paulo SP Brazil; ^5^ Departament of Biosciences Universidade Federal de São Paulo Santos SP Brazil; ^6^ Department of Medicine Escola Paulista de Medicina Universidade Federal de São Paulo São Paulo SP Brazil; ^7^ Department of Microbiology Immunology and Parasitology Escola Paulista de Medicina Universidade Federal de São Paulo São Paulo SP Brazil; ^8^ Cambridge Institute for Medical Research Cambridge UK

**Keywords:** 17β‐estradiol, COVID‐19, estrogen receptors, gender, SARS‐CoV‐2, VERO E6 cells

## Abstract

The COVID‐19 has originated from Wuhan, China, in December 2019 and has been affecting the public health system, society, and economy in an unheard‐of manner. There is no specific treatment or vaccine available for COVID‐19. Previous data showed that men are more affected than women by COVID‐19, then we hypothesized whether sex hormones could be protecting the female organism against the infection. VERO E6 cells have been commonly used as in vitro model for SARS‐CoV‐2 infection. In our experimental approach, we have treated VERO E6 cells with 17β‐estradiol to evaluate the modulation of SARS‐CoV‐2 infection in this cell line. Here we demonstrated that estrogen protein receptors ERα, ERβ, and GPER1 are expressed by VERO E6 cells and could be used to study the effects of this steroid hormone. Previous and 24‐hours post‐infection, cells treated with 17β‐estradiol revealed a reduction in the viral load. Afterward, we found that SARS‐CoV‐2 infection per se results in ACE2 and TMPRSS2 increased gene expression in VERO E6‐cell, which could be generating a cycle of virus infection in host cells. The estrogen treatment reduces the levels of the TMPRSS2, which are involved with SARS‐CoV‐2 infectiveness capacity, and hence, reducing the pathogenicity/genesis. These data suggest that estrogen could be a potential therapeutic target promoting cell protection against SARS‐CoV‐2. This opens new possibilities for further studies on 17β‐estradiol in human cell lines infected by SARS‐CoV‐2 and at least in part, explain why men developed a more severe COVID‐19 compared to women.

## Graphical abstract

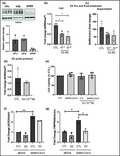

**Key points summary**

- Epidemiologic data show a greater mortality rate in men over women.
- One hypothesis is that female hormones may play a role in the disease onset.
- The results show that 17β‐estradiol can reduce SARS‐CoV‐2 infection in vitro.
- Additionally, estrogen can reduce TMPRSS2 gene expression after SARS‐CoV‐2 infection, which is involved with coronavirus infectiveness capacity.
- This data can contribute to better understand the role of estrogen in COVID‐19, which can open new therapeutic possibilities.

## 1 INTRODUCTION

COVID‐19 became the biggest pandemic of this century and could be possibly considered the most devastating, after the Spanish flu. Despite we had the previous sort of pandemic coronaviruses [e.g., SARS‐CoV (severe acute respiratory syndrome coronavirus) and MERS‐CoV (Middle East respiratory syndrome coronavirus)], they share some pathogenic mechanisms, but some of them are not completely understood. The scientific efforts to produce knowledge on COVID‐19 are not following the velocity of worldwide spreading of SARS‐CoV‐2 and, consequently, accumulating more than 22 million people infected until now (https://coronavirus.jhu.edu/map.html).

Epidemiologic data show a greater mortality rate in men over women (https://globalhealth5050.org/covid19/age‐and‐sex‐data/), and one hypothesis is that female hormones may play a role in the disease onset. For instance, Ding et al. (2020) collected important evidence on sex differences in COVID‐19, observing that female patients have a better prognosis than males in a multi‐hospital study (Ding et al., 2020). Accordingly, they have correlated those differences with the levels of sex hormones and concluded that in non‐menopausal patients the 17β‐estradiol levels are negatively correlated with pro‐inflammatory interleukins (IL‐6, IL‐8, IL2 receptor) and tumor necrosis factor‐alpha (TNF‐α), suggesting a modulation of immune response by estrogen (Ding et al., 2020). Estrogens have genomic and non‐genomic actions, and activate nuclear estrogen receptor alpha (ERα), nuclear estrogen receptor beta (ERβ) and membrane G‐protein‐coupled estrogen receptor (GPER1). These pathways have been associated with several anabolic protective actions in different organs, including lungs (for review see [Tofovic & Jackson, 2019]), cardiovascular system (for review see [Iorga et al., 2017]) and immune responses course (Di Florio et al., 2020).

One of the most used in vitro models for studying viral infections, including human‐ coronaviruses, is the monkey kidney VERO E6 cell line. This cell line has a good infective rate for SARS‐CoV‐2 and has the appropriate intracellular machinery to replicate this virus (Qinfen et al., 2004). Other than coronaviruses, studies with Zika virus have been performed successfully in this cell line, which qualifies this lineage as a versatile model for viral infection studies (Barreto‐Vieira et al., 2017). SARS‐CoV‐2 uses ACE2 (Angiotensin Converting Enzyme 2), as the main host cell surface recognition pattern and serine proteases such as TMPRSS2 (Transmembrane Protease Serine 2) for cell entry. Both are critical to cellular infection and are constitutively membrane‐bound proteins present in VERO E6 cells (Hoffmann et al., 2020). Recently it has emerged as a good biological model for translating the promising pharmacological and therapeutic strategies for COVID‐19, since there are limitations in the use of animal models for drug testing. Many drugs proposed to treat COVID‐19 were tested first in VERO E6 cells such as Remdesivir and Chloroquine, which has shown that the associative therapy could work on SARS‐CoV‐2 infection (Wang, Cao, et al., 2020). Another antiviral, Favipiravir, which blocks the RNA‐dependent RNA polymerase (RdRp), showed a protective effect in mice against the Ebola virus, and has its efficacy firstly proved in VERO E6 cells (Oestereich et al., 2014).

Taking into account that the COVID‐19 pandemic killed more than 1.5 million people in the world, affecting the health system, economy, and society in general, a quick response to the society regarding pharmacological therapeutic strategies is urgently required. Many drug trials showed good results in cell models as chloroquine (by blocking virus uncoating upon lysosome basification), but systemically has some drawbacks, such as arrhythmia and cardiac arrest (Mubagwa, 2020). Therefore, sex hormone‐related therapy may come as a promising pharmacological strategy due to its minimal collateral effects if adopted in short‐term. Hence, Glinsky (2020) analyzed several data based on protein‐protein interaction (PPI) network interactome map produced with SARS‐CoV‐2 proteins in human HEK‐293 cells (Gordon et al., 2020) and noticed that estrogen have the potential to modulate the expression of 61% of proteins related to virus‐human protein interactome (Glinsky, 2020), suggesting that this hormone could be associated to human cell protection from infection. In the present study, we tested the efficacy and safety of 17β‐estradiol for reducing SARS‐CoV‐2 infection and/or replication in a cellular lineage model.

## 2 METHODS

The VERO E6 cells (ATCC® CRL‐1586™) cell line was cultured in Dulbecco's Modified Eagle Medium/Nutrient Mixture F‐12 (DMEM‐12; Sigma‐Aldrich, USA), phenol red‐free, with 10% fetal bovine serum (FBS; Gibco, USA), at 37 °C and 5% CO_2_ atmosphere. The Institutional Ethics Committee has approved all procedures (CEP‐UNIFESP 6864310320).

The SARS‐CoV‐2 were isolated from the nasopharyngeal sample of Brazilian patient (EPI_ISL_413016), kindly provided by Prof. Edison Durigon and Paolo Zanotto—University of São Paulo—SARS‐CoV‐2 sequenced (GenBank accession no. MT 126808) SARS‐CoV‐2 was isolated and amplified in the fourth passage in Vero E6 cells, and virus infectivity capacity was determined by plaque assay as described (Cugola et al., 2016). A titer of 5 × 10^7^ plaque‐forming units (PFU/mL) was obtained and stocked at −70°C.

First, protein expression of the estrogen receptors: ERα, ERβ, and GPER1 in VERO E6 cells were characterized by immunoblotting. 3 x 10^5^ cells of cultured VERO E6 cells were incubated for 48 hours at 5% CO2 and 37˚C. After incubation, cells were processed with lysis buffer (10 mM Tris, 10 mM NaCl, 3 mM MgCl2, 1 mM EGTA, 10% glycerol, 1% de Triton X‐100, pH 7.4) containing proteases and phosphatases inhibitors (protease inhibitor cocktail (1:100), 0.5 mM PMSF, 5 mM sodium fluoride, 0.5 mM sodium orthovanadate, and 1 mM sodium molybdate; Sigma‐Aldrich). Twenty μg of protein were loaded into a 12% SDS‐PAGE, transferred into a PVDF membrane (Millipore, USA), and immunoblotting for ERα (MC‐20, sc‐542, Santa Cruz Biotechnology Inc., 1:1000), ERβ (MC10, Thermo Fisher Scientific, 1:2000), and GPER1(ab39742, Abcam, 1:1000) were performed at 4°C overnight. Protein load was normalized by anti‐GAPDH (G8795, Sigma‐Aldrich, 1:5000, incubated for 2 hours at room temperature). The secondary antibodies used were anti‐rabbit (A0545, Sigma‐Aldrich, 1:5000; for GPER1) and anti‐mouse (A9044, Sigma‐Aldrich, 1:5000; for ERα, ERβ, and GAPDH), incubated for 1 hour at room temperature. The chemiluminescence reaction was developed with ECL (Perkin‐Elmer, USA), photodocumented in UVTEC (Cambridge, UK) and bands analyzed with ImageJ software (NIH, USA).

Referred to SARS‐CoV‐2 infection, 1 x 10^5^ of cultured VERO E6 cells were previously treated with 17β‐estradiol (E2) at 10^−9 ^M (Tocris Bioscience) during 24 hours at 5% CO2 and 37˚C. DMSO (at concentration 0.00001%) was used in parallel as vehicle control (CTR group). The concentration of 17β‐estradiol range (10^−9^ and 10^−7 ^M) was adopted based on our previous data in other cell lines (data not shown). For infection, estimated MOI (multiplicity of infection) of 0.2 of SARS‐CoV‐2, were diluted in 200 µL of DMEM F12 with 1% FBS and added or not (mock) to each treated cell‐well condition and incubated for 2 hours at 5% CO_2_ and 37˚C. Afterward, the cells were rinsed with PBS and treated for additional 24 hours with 17β‐estradiol at the same previous conditions. The duration (24 hours) of treatment protocol of SARS‐CoV‐2 infected cells was chosen based on recent literature data (Hoffmann et al., 2020). Additionally, in order to evaluate the direct effect of 17β‐estradiol on viral cell entry, another set of experiments was performed, called “pulse protocol.” In this protocol, 17β‐estradiol (10^−9^ and 10^−7 ^M), was added to VERO E6 (1 x 10^5^ cells /well) concomitantly to SARS‐CoV‐2 (MOI 0.2, diluted in DMEM‐F12 with 1% FBS) infection during 2 hours (at 5% CO_2_ and 37˚C). Thereafter, cells were rinsed with PBS, and incubated for additional 24 hours in DMEM‐F12 with 10% FBS, without 17β‐estradiol.

Then, both plated cells and supernatant of all experiments performed were harvested and had their RNA extracted by RNeasy Mini Kit (Qiagen, USA – for cell extracts) and Quick RNA viral kit (Zymo Research, USA – for supernatant extracts). The virus was detected by quantitative reverse‐transcriptase polymerase chain reaction (RT‐qPCR) following the protocol described by Corman et al. (2020), using AgPath‐ID One Step kit (Applied Biosystems, USA), in a 7500 Real‐Time PCR Instrument (Applied Biosystems, USA). The following primers and probes sequences were applied: SARS‐CoV‐2 nucleocapsid (N) primer Fwd 5’ CACATTGGCACCCGCAATC 3’; primer Rv 5’ GAGGAACGAGAAGAGGCTTG 3’; Probe 5’ FAM‐ACTTCCTCAAGGAACAACATTGCCA‐BBQ 3’. RNAse P primer Fw 5’ AGATTTGGACCTGCGAGC 3’; primer Rv 5’ GAGCGGCTGTCTCCACAAGT 3’; Probe 5’ FAM‐TTCTGACCTGAAGGCTCTGCGCG‐BHQ; was used as housekeeping gene on cellular RNA viral analysis, using the relative comparison method (ΔΔCT). For the analysis, it was used 7500 Software v2.0.6. Viral RNA supernatant levels were expressed in PFU/mL, the real‐time PCR of each sample was compared with threshold cycle (Ct) value in the SARS‐CoV‐2 standard curve for nucleocapsid (N), which was obtained carrying out a 10‐fold serial dilution of the virus titrated sample. For the comparison of viral load among groups, the SARS‐CoV‐2 nucleocapsid (*N*) mRNA was normalized by the expression of the housekeeping gene *RNAseP* (referred as *N*/*RNAseP*), and data were presented as control (CTR) fold change.

Cellular gene expression was quantified by RT‐qPCR presented in ΔΔCT using *ACTIN* gene as housekeeping to normalize the absolute quantification. For *ACE2* and *TMPRSS2* mRNA quantification, the cDNA was produced with a High Capacity Kit (Applied Biosystems, USA) and the RT‐qPCR reaction was performed by the SYBR‐Green protocol. The following primers were used: *TMPRSS2* primer Fwd 5’CTC TAA CTG GTG CGA TGG CG3’; primer Rv 5’ TGCCAGGACTTCCTCTGAGATG 3’. *ACE2* primer Fwd 5’ CATTGGAGCAAGTGTTGGATCTT 3’; primer Rv 5’ GAGCTAATGCATG CCATTCTCA 3’. *ACTIN* primer Fwd 5’ CAGAGCCTCGCCTTTGCCGATCC 3’; primer Rv 5’ GACGACGAGCGCGGCGATATCA 3’. The analysis was performed using ΔΔCT and the samples were compared to the internal *ACTIN* control.

The cellular viability of infected or 17β‐estradiol‐treated cells was assessed by MTT reagent (Sigma Aldrich). By the end of each incubation (24 hours), MTT solution (5 mg/mL) was added per well at 0.5% and after 4 hours, formazan crystals were solubilized with isopropyl alcohol. Sample absorbance values were measured using a spectrophotometer Ascent (Thermo scientific), and cellular viability was estimated according to the amount of formazan. Data were showed considering control as 100% viability.

All experiments were conducted using two technical duplicates, and for estrogen treatments using at least two independent experiments (N = 3). The Student t‐test (for two‐group comparison) and one‐way ANOVA (for multiple comparisons) was performed using Prism 8.0 software (GraphPad, USA), and considered as significant *p* < 0.05.

## 3 RESULTS

### 3.1 Estrogen receptors protein expression in VERO E6 cells

**FIGURE 1:**
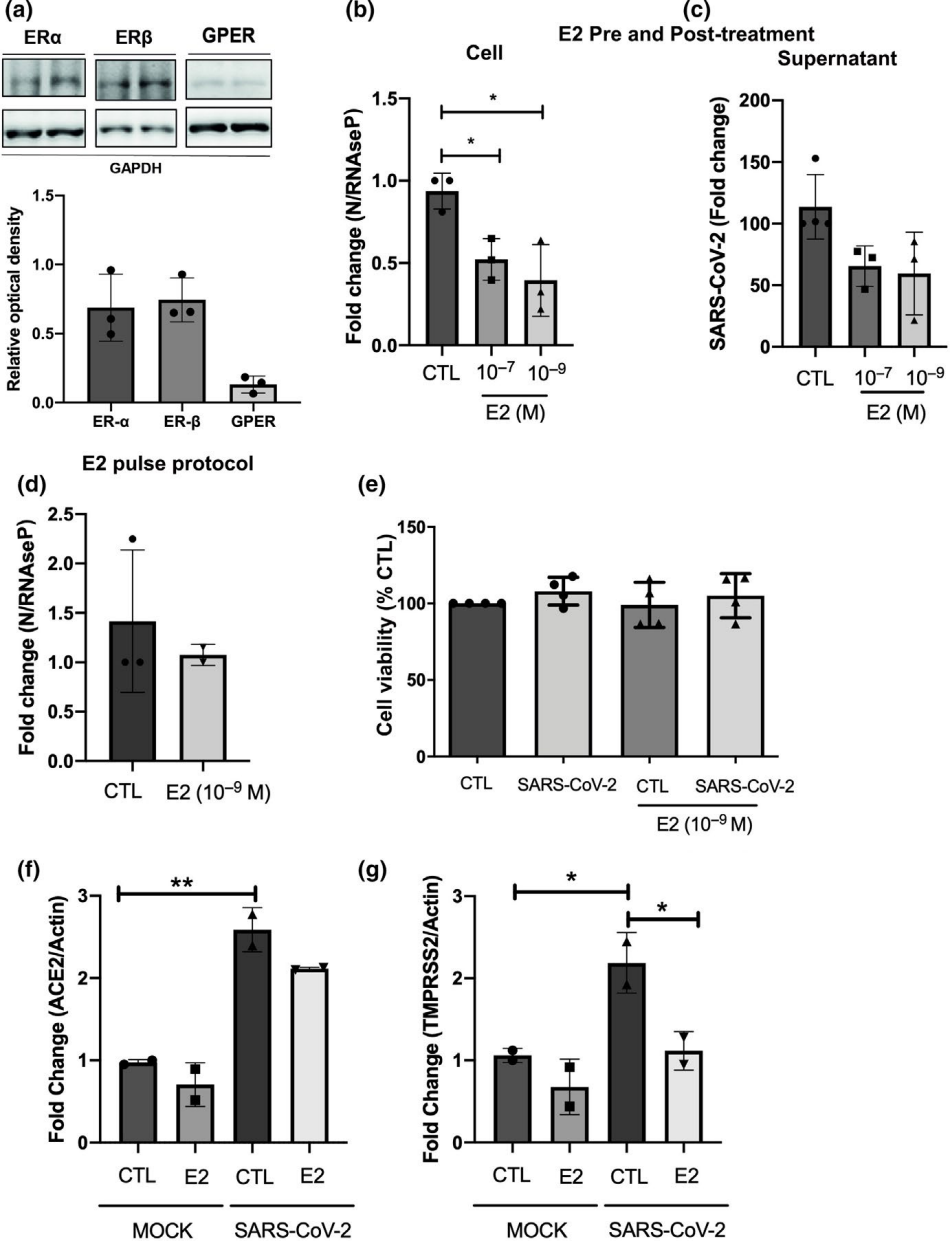
17β‐estradiol protects cells against SARS‐CoV‐2 infection/replication, modulating the mRNA levels of *TMPRSS2*. (a) Protein expression by the Western blotting of estrogen receptors ERα, ERβ, and GPER1 in VERO E6 cells. (b) SARS‐CoV‐2 content in VERO E6 cellular extracts, quantified by RT‐qPCR, after 24 hour‐treatment with two concentrations of 17β‐estradiol (E2), showing that both are effective in reducing viral load. (c) SARS‐CoV‐2 released in the supernatant after 24 hour‐treatment treatment with E2. (d) Virus pretreatment with E2 and during “pulse protocol” (for 2 hours, during virus infection) and quantifications of SARS‐CoV‐2 in cellular extracts. (e) MTT assay of VERO E6 cells infected or not with SARS‐CoV‐2 and treated or not with 10^−9 ^M of E2. (f,g) Gene expression of *ACE2* (f) and *TMPRSS2* (g) before and after SARS‐CoV‐2 infection, demonstrating that virus elevates the gene expression and the treatment with E2 downregulates the *TMPRSS2*. In b, c, e, f, and g the VERO E6 cells were pre‐treated for 24 h with 17β‐estradiol, before virus infection, and then post‐treated for additional 24 h. * considered as significant *p* < 0.05. For (b–g) *N* = 2–3.

Initially, to verify whether VERO E6 could be a suitable model for 17β‐estradiol treatment, we characterized the estrogen receptors in this cell line by immunoblotting. Data shown in Figure 1a demonstrate that VERO E6 cells normally express both nuclear (ERα, ERβ) and membrane estrogen receptors (GPER1).

### 3.2 Effects of 17β‐estradiol on SARS‐COV‐2 infection

Accordingly, we tested the effects of 17β‐estradiol on SARS‐CoV‐2 infection or virus replication in the cell line. Both 17β‐estradiol 10^−7^ and 10^−9 ^M presented a reduction in cellular viral load (Figure 1b) after 24 hours post‐infection, although the virus released in the supernatant did not differ between control and estrogen‐treated groups (Figure 1c). Figure 1b presents data showing that 17β‐estradiol was able to reduce significantly over 40% (*p* < 0.05) of cellular virus load.

Additionally, no potentially direct effect of 17β‐estradiol on the virus entrance was observed, since 17β‐estradiol (10^−9 ^M) treatment concomitantly with SARS‐CoV‐2 (MOI 0.2) infection showed no difference of the SARS‐CoV‐2 viral load after 24 hours of infection in “pulse protocol” (Figure 1d). Noteworthy, neither the SARS‐CoV‐2 infection nor the concentrations of 10^−9 ^M of 17β‐estradiol used promoted any significant alterations in VERO E6 viability (Figure 1e).

### 3.3 17β‐estradiol changes the expression of SARS‐COV‐2 cellular receptors

After virus infection, we also measured the levels of *ACE2* and *TMPRSS2* in the VERO E6 cell line. The data show that SARS‐CoV‐2 infection *per se* is capable of enhancing significantly the *ACE2* (Figure 1f) (*p* < 0.05) and *TMPRSS2* mRNA gene expression (Figure 1g) (*p* < 0.05), whereas the pre‐treatment with 17β‐estradiol significantly downregulated *TMPRSS2* mRNA gene expression (*p* < 0.05).

## 4 DISCUSSION

The influence of sex hormones in studies of viral infection has been consolidated over the years (Huber & Pfaeffle, 1994; Reading et al., 2003; Torcia et al., 2012). Evidence of immune response modulation by ERα and ERβ mainly on Toll‐like receptors (TLR) 4, 7, 8, and 9 signaling (Rizzetto et al., 2018), highlight a potential activity of 17β‐estradiol on SARS‐CoV‐2 infection, as its single‐stranded viral RNA carrier, can be detected by TLR7 and TLR8 (Sallenave & Guillot, 2020). Knowing that, the present work demonstrates the potential ability of 17β‐estradiol to modulate SARS‐CoV‐2 infection.

Working as main receptors during estrogen signaling, ERα, ERβ, and GPER1 protein expression were initially verified in our in vitro cell model and we showed that VERO E6 cells express proteins estrogen receptors. This data corroborates previous findings that have shown the presence of ERα and ERβ in this cell line, and it was used to test estrogen receptor modulators against Ebola virus infection in vitro (Johansen et al., 2013).

Considering that the stimulation of estrogen receptors demands the recruitment of complex transcriptional machinery for estrogenic genomic actions, we tested the effects of two 17β‐estradiol doses on SARS‐CoV‐2 infection. Data showed that both concentrations of 17β‐estradiol reduced the cellular viral load, compared to untreated control. Affinity and consequent sensitization of estrogen receptors to 17β‐estradiol are inversely proportional to dose and it is known that concentrations above 10^−7 ^M can down‐regulate both ERα and ERβ (Lucas et al., 2010).

Estrogen can potentially participate in a many of cell protection against virus infection. For example, by the SARS‐CoV adapted for mouse studies (MA15), Channappanavar et al. (2017) infected males and females mice to explore the role of sex hormones in animal survival rate (Channappanavar et al., 2017). This group showed that ovariectomized female mice had a more severe form of the disease compared to control, and ICI (fulvestrant, an estrogen nuclear receptor antagonist) was able to reduce the survival rate in females. Moreover, castrated male mice did not show an increase in death number due to SARS‐CoV infection compared to the control group, suggesting that the susceptibility to SARS‐CoV severity could be sex‐related, and estrogen may play an important role in the disease onset. Adding another perspective, it has been demonstrated that 17β‐estradiol interferes with hepatitis C life cycle (Magri et al., 2017), suggesting a direct mechanism of steroid action on virus, rather than only by modulating host intracellular homeostatic processes. Moreover, sterols have been suggested to potentially modify virus infection capacity (Rezanka et al., 2009). Thus, considering that the pre‐treatment with estrogen has been demonstrated to promote protective actions in acute lung injury, due to the preventive anti‐inflammatory effects (Fantozzi et al., 2018), it might be of interest to investigate the role of this hormone in COVID‐19.

After virus infection, we also measured the levels of *ACE2* and *TMPRSS2* in the VERO E6 cell line. The data show that SARS‐CoV‐2 infection *per se* is capable of enhancing the *ACE2* and *TMPRSS2* mRNA gene expression. Many reports bring the view that ACE2 is important for host cell recognition, and TMPRSS2 participates in activating coronavirus Spike protein (Hoffmann et al., 2020) (Glowacka et al., 2011). Matsuyama et al. (2020) generated VERO E6 overexpressing TMPRSS2 and found that SARS‐CoV‐2 RNA in supernatants were more than 100 times higher than control cells, suggesting a critical role of this serine protease in the virus infectivity capacity (Matsuyama et al., 2020). Moreover, the role of estrogen receptors in SARS‐CoV‐2 infection and mechanism has not been explored yet, although these results suggest that estrogen signaling is critical for virus cellular protection. It is noteworthy that a recent paper showed the ability of 17β‐estradiol to modulate *ACE2* gene expression levels, reinforcing that the role of sex hormones should be further explored in COVID‐19 (Stelzig et al., 2020).

In the present work, we infected cells with SARS‐CoV‐2 and our data suggest that the downregulation in *TMPRSS2* expression (Figure 1g) could be a consequence of the activation of estrogen receptors in VERO E6 cells, and we suggest that this could be related to the reduction of virus content inside the cells (Figure 1b). This serine protease is critical for the virus entry in mammalian cells (Hoffmann et al., 2020), and previous data suggest that estrogen treatment can modulate the levels in MCF‐7 ER‐positive cells (Wang, Dhindsa, et al., 2020). Regarding the infection‐prone proteins, Stelzig et al. (2020) treated a pulmonary NHBE cell line with 17β‐estradiol for 24 hours and observed a reduction in *ACE2* gene expression, but not in *TMPRSS2* (Stelzig et al., 2020). Here we found that 17β‐estradiol maintained in cell culture for 48 hours—in non‐infected VERO E6 cells (mock group)—did not promote alterations in *ACE2* and *TMPRSS2* gene expression (Figure 1f,g – mock group). The differences observed in Stelzig work could be explained by fluctuations in the gene expression over time, since we adopted a short‐term 17β‐estradiol treatment in our protocol. In addition, variations in gene expression might not necessarily correspond to the protein expression pattern. Although no significant difference was found in *ACE2* mRNA modulation by 17β‐estradiol treatment in non‐infected cells (mock group – Figure 1f), VERO E6 cells efficiently responded to estrogen treatment by reducing SARS‐CoV‐2 load in cells (Figure 1b). In addition, in this work, we proposed to initiate the study of the 17β‐estradiol mechanism of action in SARS‐CoV‐2 infection, and we recognize that further investigations are necessary to establish the link between the reduction in viral load and TMPRSS2 expression by estrogen treatment. Finally, the pre‐treatment with 17β‐estradiol of isolated virus (“pulse protocol”) caused no significant differences, suggesting the lack of a direct effect of this hormone on the SARS‐CoV‐2 before cell infection. However, our results indicate that estrogen is not interfering with the SARS‐CoV‐2 infection capacity of the VERO E6 cells (Figure 1d), suggesting that estrogen could act in the genomic host‐response pathway. These results confirm what was suggested in our previous publication about the effects of estrogen receptors on SARS‐CoV‐2 infection (Stilhano et al., 2020).

## 5 CONCLUSION

Overall, since estrogen is a naturally occurring hormone in humans, with low adverse effects under short‐term usage, this data indicate that estrogen is capable of reducing SARS‐CoV‐2 infection in vitro. This data can help to understand why men are more susceptible to severe forms of COVID‐19 than woman. Moreover, in accordance with the previous data that highlights several aspects of the possible role of estrogen receptors in the COVID‐19 disease progression, we suggest that this could be potentially tested in human cell lines and animal models, prior to human use for COVID‐19. Therefore, it might be relevant to consider in future studies that hormone therapy may act as an adjuvant in pharmacologic antiviral therapies, especially considering estrogen pre‐treatment, which has been demonstrated to play a role in systemic response to pathogens and in the inflammatory system.

## CONFLICT OF INTERESTS

The authors declare that no competing interests and conflicts of interest in this article.

## AUTHORS’ CONTRIBUTION

Conception or design of the work: RPU, RSS, CMP, LMRJ, RMRL. Acquisition, analysis or interpretation of the data for the work: RPU, RSS, CMP, LMRJ, JTM, ALR, EFC, CTB, RMBM, SSS, GJSP, MSN, TBB, CSB, AJC, RMRL. Drafting the work or revising it critically for important intellectual content: RPU, RSS, CMP, LMRJ, JTM, ALR, EFC, CTB, RMBM, SSS, GJSP, MSN, TBB, CSB, AJC, RMRL. All authors approved the final version of the manuscript; agree to be accountable for all aspects of the work in ensuring that questions related to the accuracy or integrity of any part of the work are appropriately investigated and resolved; all persons designated as authors qualify for authorship, and all those who qualify for authorship are listed.

## Data Availability

Data available on request from the authors.
